# Effects of self-discrepancy on emotional information processing in competitive tennis players

**DOI:** 10.3389/fpsyg.2025.1730711

**Published:** 2026-01-12

**Authors:** Xiaozhong Su, Rong Shangguan, Meiji Luo

**Affiliations:** 1 School of Educational Sciences, Hunan Normal University, Changsha, China; 2 School of Sports and Music, Central South University of Forestry and Technology, Changsha, China; 3 College of Physical Education, Hunan Normal University, Changsha, China; 4 Hengyang No.20 Middle School, Hengyang, China

**Keywords:** attentional bias, emotional information processing, self-discrepancy, self-referential processing, tennis players

## Abstract

**Introduction:**

This study examined how self-discrepancy levels shape emotional information processing in competitive tennis players. Using a self-referential paradigm and a key-press/spatial-cueing task, we tested whether self-discrepancy biases the representation of valenced self-information and whether this negativity generalizes to attentional operations (spatial orienting/disengagement).

**Methods:**

A two-step design was adopted. A pilot phase was used only for participant screening/grouping and stimulus quality control (no inferential testing). In Experiment 1, a between-subjects design assessed athletes with high vs. low self-discrepancy across self-type (ideal/ought/actual) and word valence (positive/negative), indexing processing speed and self-descriptive endorsement. In Experiment 2, a mixed design tested discrepancy level (high/low; between-subjects) and emotion type (positive/negative/neutral) and cue type (valid/invalid; within-subjects) to quantify emotional processing speed and attentional bias (vigilance/disengagement).

**Results:**

In Experiment 1, high self-discrepancy athletes responded faster to negative words, whereas low self-discrepancy athletes responded faster to positive words; high self-discrepancy athletes also endorsed more negative self-descriptors. In Experiment 2, high self-discrepancy athletes responded faster to negative stimuli, slower to positive stimuli, and showed no reliable differences for neutral stimuli, consistent with a discrepancy-linked negativity extending from lexical self-reference to attentional processing.

**Discussion:**

Findings suggest that elevated self-discrepancy increases the accessibility and endorsement of negative self-referent content and may predispose athletes to negative cognitive schemas, yielding accelerated processing of negative information and stronger attentional bias toward negative stimuli. This work highlights the importance of individual differences in self-cognition for emotional regulation and offers a mechanistic bridge from self-structure to attentional bias in sports psychology.

## 1 Introduction

Performance at critical moments frequently determines the outcome of a tennis match. Athletes' retrospective accounts commonly emphasize perceived success or failure under pressure—for example, claiming they “handled the big points well” or that they “choked” when it mattered. When an athlete perceives a gap between actual performance (the actual self) and internal standards (the ideal or ought self), strong negative affect (e.g., frustration, anxiety) and maladaptive behaviors (e.g., racket-throwing) may follow, with clear implications for subsequent performance. For instance, during the ATP Cup, Stefanos Tsitsipas lost emotional control after repeated double faults, while in a comparable situation at the Australian Open Li Na reportedly appraised her errors more rationally and adjusted her strategy. These contrasting responses raise an important question: why do athletes respond so differently to similar performance setbacks?

Self-Discrepancy Theory (SDT) provides a parsimonious account. SDT distinguishes three self-representations—ideal, ought, and actual—where the actual self reflects how individuals perceive their current attributes (“who I am now”), the ideal self represents hopes, wishes, and aspirations (“who I would like to be”), and the ought self reflects perceived duties, responsibilities, and obligations imposed by oneself or significant others (“who I should be”) (Gürcan-Yıldırnm and Gençöz, 2022; Heron and Smyth, 2013). SDT posits that discrepancies between actual and ideal selves (actual–ideal, AI) and between actual and ought selves (actual–ought, AO) give rise to different affective profiles. Specifically, AI discrepancies are typically associated with dejection-related affect (e.g., disappointment, sadness), whereas AO discrepancies are associated with agitation-related affect (e.g., anxiety, guilt) that stems from concern about negative outcomes (Higgins, 1987; Hu et al., 2022; Oh et al., 2023; Martín-García et al., 2024). Beyond their emotional consequences, self-discrepancies, as relatively stable cognitive structures, shape how individuals allocate attention, process information, and regulate motivation (Higgins, 1987; Yang, 2015; Hu et al., 2022).

Self-referential processing refers to the cognitive process through which individuals evaluate, encode, and integrate information in relation to their self-representations (Sui and Humphreys, 2016). During this process, self-related information is typically processed more deeply and remembered more accurately than other-related information (known as the self-reference effect) (Yaoi et al., 2015; Brèdart, 2016), as it is more strongly connected to pre-existing self-schemas. When individuals experience self-discrepancies (e.g., between the actual and ideal/ought selves), specific self-representations and associated emotions such as disappointment or anxiety are more easily activated. This activation biases attentional and cognitive resources toward information that is consistent with the activated self-representation. In other words, self-discrepancy functions not only as an emotional vulnerability factor but also as a cognitive “filter” that determines which types of self-content receive preferential processing, thereby influencing attention allocation, encoding speed, and evaluative tendencies (Kujawa et al., 2022; Zhao et al., 2023, 2024).

Based on this mechanism, we hypothesize that athletes with high self-discrepancy would respond faster and more frequently to negative self-descriptive words in self-referential tasks—reflecting stronger negative self-associations—and show attentional bias toward negative cues in dot-probe tasks. Therefore, the present study adopted self-referential encoding and attention paradigms to directly link self-discrepancy (as an underlying self-schema) with self-referential processing and early attentional dynamics, providing a clearer framework for understanding how self-discrepancy influences emotional information processing and attentional bias in athletes.

In competitive sport—an environment characterized by frequent evaluative feedback and acute performance pressure—self-discrepancies may bias attentional allocation toward performance-relevant, negatively valenced cues (e.g., a coach's stern look, crowd reactions, or replayed errors). Such attentional biases may amplify negative affect and interfere with task-relevant processing, thereby degrading technical execution (Xu et al., 2022; Jin and He, 2021). Although research in non-athlete populations has documented links between self-discrepancy and cognitive biases in attention and memory (Hu et al., 2022; Zhao et al., 2024), little is known about how AI and AO operate within athlete populations—particularly regarding the behavioral patterns of emotional information processing and attention bias during competition.

To address this gap, the present study examines how self-discrepancy manifests in competitive tennis players and whether distinct types or magnitudes of self-discrepancy are associated with systematic biases in emotional information processing. We adopt a multilevel experimental approach that probes both self-referential processing and attentional dynamics. Experiment 1 employs the Self-Referential Encoding Task (SRET) to characterize self-associations via endorsement rates and reaction times to valenced trait words, thereby revealing how AI and AO relate to self-representational processing (Zhao et al., 2024; Kujawa et al., 2022). Experiment 2 uses the Dot-Probe Paradigm to quantify attentional orienting and disengagement toward positive, neutral, and negative stimuli, producing behavioral indices of vigilance and disengagement costs (Perera et al., 2025). Together, these paradigms allow us to link self-representational biases to early attentional processes that are plausibly implicated in in-match emotion regulation and performance.

Building on SDT and emotion–attention research, we pose three primary research questions and hypotheses. Prior work shows that self-discrepancies are linked to negative self-referent cognition and symptoms (Higgins, 1987; Hu et al., 2022; Oh et al., 2023; Martín-García et al., 2024), biased processing of self-relevant emotional information (Kujawa et al., 2022; Zhao et al., 2023), and vulnerability to anxiety and dysregulation in performance contexts (Martín-García et al., 2024; Oh et al., 2023). In parallel, attentional-bias studies indicate vigilance for and difficulty disengaging from negative cues in anxious or threat-sensitive individuals (Bar-Haim et al., 2007; Mogg and Bradley, 1998; Heeren et al., 2015; Bryant, 2021), including athletes under competitive stress (Rahimi et al., 2022; Zhao et al., 2025; MacLeod et al., 1986). Against this backdrop, our hypotheses extend SDT from self-referential content to attentional dynamics in sport.

RQ1 (Self-representational signatures): What behavioral signatures of self-discrepancy emerge during self-referential processing?

Given evidence that self-discrepancies amplify access to negative self-beliefs and dejection/agitational affect (Higgins, 1987; Hu et al., 2022; Yang, 2015), we expected systematic differences in endorsing and processing valenced self-descriptors.

H1: Compared with athletes low in self-discrepancy, athletes high in AI or AO will (a) endorse more negative/self-discrepant items and (b) show faster reaction times to negative self-referential items, indicating stronger negative self-associations.

RQ2 (Attentional bias): Do athletes with differing AI/AO profiles exhibit systematic attentional biases toward emotional stimuli?

Building on meta-analytic work on threat-related attentional bias and self-referential processing (Bar-Haim et al., 2007; Kujawa et al., 2022; Zhao et al., 2024), we differentiated predictions for AO- vs. AI-dominant discrepancies.

H2a: Athletes high in AO will demonstrate vigilance toward negative stimuli and/or greater difficulty disengaging from negative stimuli (i.e., faster orienting to negative cues and larger disengagement costs).

H2b: Athletes high in AI will show reduced responsiveness to positive stimuli and/or sustained processing of negative stimuli (e.g., attenuated positive bias or slower recovery following negative cues).

RQ3 (Processing components and ecological relevance): If attentional biases are present, which processing components (orienting vs. disengagement vs. cue-congruency effects) underlie them, and do laboratory bias indices predict on-court emotional dysregulation or performance decrements under pressure?

Attentional-control models and sport research highlight disengagement from threat and regulation under pressure as key mechanisms linking cognition to performance (Eysenck et al., 2007; Rahimi et al., 2022; Prior et al., 2025).

H3a: High AO athletes will show larger congruency × group interactions—particularly greater reaction-time costs in incongruent trials—consistent with impaired disengagement from negative stimuli.

H3b: Greater negative attentional biases, especially disengagement costs, will be associated with higher incidence of on-court emotional dysregulation and poorer performance on critical points.

To test these hypotheses, AI and AO will be operationalized using established scoring procedures for self-discrepancy paradigms (e.g., Gabel et al., 2024; Kujawa et al., 2022). Primary dependent measures will include SRET endorsement counts and reaction times, and dot-probe bias indices (orienting, disengagement, congruency effects). Mixed-design ANOVAs and hierarchical regression models will be used, with trait anxiety, depressive symptoms, competitive level, years of training, and self-efficacy entered as covariates where appropriate.

## 2 Pilot study: investigating self-discrepancy in adolescent tennis players

### 2.1 Method

#### 2.1.1 Participants

A total of 70 participants were recruited for the pilot study (31 females). The mean age of the sample was *M* = 19.76 ± 1.86 years. All participants were right-handed, had normal or corrected-to-normal vision, and had no history or family history of *major* psychiatric disorders. For the purposes of the pilot study, these participants were used to compute individual self-discrepancy indices and to screen and assign athletes to the high- vs. low-discrepancy groups that would be used in Experiment 1.

In addition, an independent sample of fifteen tennis players from Hunan Province (ITN 3.5–5.0) participated in the stimulus evaluation component of the pilot study. This separate material-evaluation sample was used exclusively to assess item-level valence and meaningfulness; it was not used for participant grouping or any hypothesis tests.

#### 2.1.2 Materials

Fifteen tennis players from Hunan Province, with skill levels ranging from ITN 3.5 to 5.0, participated in the pilot assessment and evaluation of the stimuli for Experiment 1. The experimental materials were selected from Anderson's word list and the Chinese affective word system developed by Wang et al. (2008). A total of 104 adjectives were selected, including 52 positive and 52 negative words, which were presented in a randomized order. Prior to Experiment 1, the independent evaluation sample rated the valence and meaningfulness of each candidate word on separate 5-point scales. These checks were used solely to finalize the stimulus set for Experiment 1.

##### 2.1.2.1 Material evaluation (quality control only)

Prior to the experiment, participants assessed the valence and meaningfulness of the selected words. Both dimensions were rated on a 5-point scale. Participants rated each word for “pleasantness” (1 = extremely unpleasant, 2 = somewhat unpleasant, 3 = neither unpleasant nor pleasant, 4 = somewhat pleasant, 5 = extremely pleasant) and “meaningfulness” (1 = not meaningful at all, 2 = slightly meaningless, 3 = neutral, 4 = somewhat meaningful, 5 = highly meaningful). Analysis of variance indicated a significant difference in valence between positive and negative words, *t*(102) = 47.85, *p* < 0.01, $\eta_{p}^{2}$ = 0.96, whereas no significant difference was found for meaningfulness, *t*(102) = 0.98, *p* = 0.33, $\eta_{p}^{2}$ = 0.009. All selected adjectives consisted of three Chinese characters. These checks informed item retention/exclusion and did not serve as hypothesis tests.

#### 2.1.3 Procedure

The experiment was conducted in a quiet, undisturbed lounge following the athletes' training or competition sessions. The experimental program was programmed using E-Prime 2.0, and stimuli were presented on a laptop monitor with a visible area of 322 mm × 213 mm, a refresh rate of 60 Hz, and a resolution of 1,024 × 768 pixels. Participants were seated approximately 60 cm from the screen.

In each trial, an adjective was presented for 1,000 ms, followed by a 300 ms presentation of a “fixation”. After the “fixation” disappeared, a self-state question appeared for 1,800 ms (e.g., “Is this your ideal self?” or “Is this your actual self?”). Participants were instructed to respond as quickly and accurately as possible by judging whether the presented word matched the specified self-condition. Responses were made using the keyboard: the “F” key indicated a match, and the “J” key indicated a non-match. Trials without a response advanced automatically after 3,000 ms.

**Figure 1:**
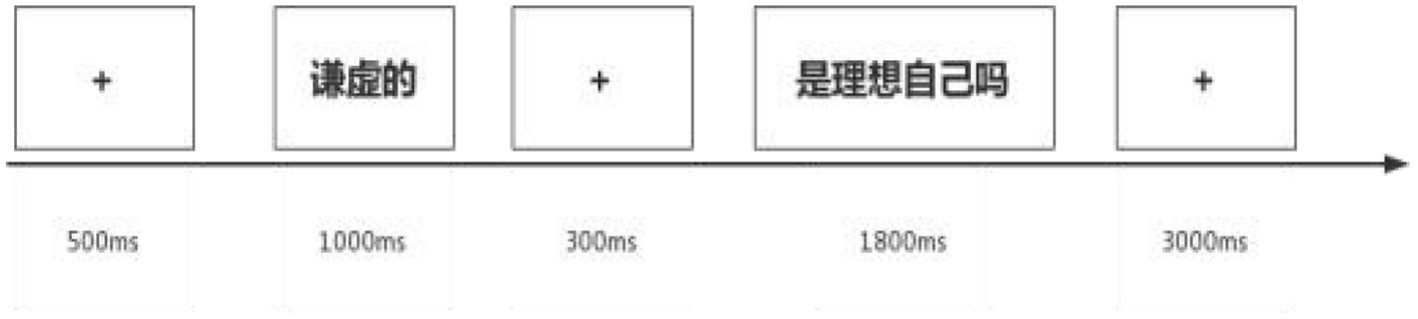
Self-referential paradigm.

This study employed a self-referential paradigm. Participants were required to judge the correspondence between the presented word and their self-concept, responding via key press. A total of 204 adjectives were presented, including four practice trials. Each adjective appeared once under the ideal self condition and once under the actual self condition, resulting in 100 trials per self-condition. The total experiment duration was approximately 20–25 min. Prior to the formal experiment, participants completed 4 practice trials using two positive and two negative adjectives that were not repeated in the main experiment (see Figure 1). The pilot task's sole purpose was to generate stable self-referential endorsement counts required to compute each participant's self-discrepancy index for grouping. No inferential comparisons between groups were conducted at this stage. The finalized stimulus set derived from the material evaluation was then used in Experiment 1.

#### 2.1.4 Behavioral data analysis and processing

Behavioral data for the self-referential paradigm were collected from 70 tennis players using a mixed-design framework: 3 (self-type: actual self, ideal self, ought self) × 2 (word valence: number of endorsed positive words, number of endorsed negative words).

The 500 ms period preceding stimulus presentation was used as a baseline. Only participants' keypress responses following stimulus presentation were collected and analyzed. All artifacts were removed, and statistical analyses of keypress responses were conducted in accordance with previous research and the objectives of the current study. No between-group or within-subject inferential tests were performed in the pilot stage; all computations supported participant inclusion/exclusion and group assignment.

The ought self is based on the responsibilities and obligations that an individual believes they should fulfill, whereas the ideal self is more conceptual and closely associated with imagined self-representations. Building on prior research, the present study primarily focused on the ideal–actual self-discrepancy. The computation method was as follows:

- Ideal self: calculated as the sum of the number of positive words endorsed and the number of negative words not endorsed under the ideal self condition. That is: ideal self = number of positive words endorsed under ideal self + number of negative words not endorsed under ideal self.
- Actual self: calculated as the sum of the number of positive words endorsed and the number of negative words endorsed under the actual self condition. That is: actual self = number of positive words endorsed under actual self + number of negative words endorsed under actual self.
- Self-discrepancy: computed as the difference between ideal self and actual self: Self-discrepancy = Ideal self – Actual self.

### 2.2 Results

This study examined tennis players' self-referential processing performance under different self-states using positive and negative words as target stimuli. A mixed analysis of variance revealed a significant main effect of self-type, *F*(2, 54) = 7.67, *p* = 0.001, $\eta_{p}^{2}$ = 0.22, and a significant main effect of word valence, *F*(1, 55) = 654.62, *p* < 0.001, $\eta_{p}^{2}$ = 0.92, with faster response times for positive words than negative words on average. Importantly, the interaction between self-type and word valence was significant, *F*(2, 54) = 38.33, *p* < 0.001, $\eta_{p}^{2}$ = 0.59.

To unpack this interaction, simple-effects analyses showed that, for the number of endorsed positive words, participants endorsed significantly more positive words under the ideal self condition compared to the ought self condition (*p* < 0.01, $\eta_{p}^{2}$ = 0.23), and also significantly more than under the actual self condition. For the number of endorsed negative words, participants selected significantly more words under the actual self condition than under the ideal or ought self conditions (*p* < 0.01, $\eta_{p}^{2}$ = 0.19), consistent with previous research (Zhang and Zhong, 2012).

These results indicate that participants' self-evaluations differed significantly across the ideal self, ought self, and actual self conditions, supporting the hypothesis that self-discrepancy exists among tennis players.

### 2.3 Discussion

Behavioral data indicated that participants under the ideal self condition tended to endorse positive words, whereas those under the actual self condition were more likely to endorse negative words. This finding is consistent with Self-Discrepancy Theory, which posits that discrepancies between an individual's ideal or ought self and their actual self give rise to self-discrepancy (Zhang and Zhong, 2012), manifesting behaviorally as biases in the selection of positive and negative words. The greater number of positive words endorsed under the ideal self condition suggests that athletes exhibit a strong positive self-cognitive tendency when evaluating themselves in terms of their ideal self. Conversely, the increased endorsement of negative words under the actual self condition reflects a certain degree of negative self-cognition in the actual self state.

Furthermore, the significant interaction between self-type and word valence indicates that self-referential processing varies across different self-states. This finding aligns with social-cognitive perspectives on self-regulation and self-evaluation (Higgins, 1987), which suggest that individuals compare and adjust among their ideal, ought, and actual selves, thereby influencing the speed of information processing and choice behavior. In the context of sports psychology, such self-discrepancies may affect athletes' emotion regulation and behavioral strategies, highlighting the importance of considering the potential impact of ideal–actual self-discrepancies on positive behavior and psychological states in training and psychological interventions.

## 3 Experiment 1: characteristics of self-referential processing in tennis players with different levels of self-discrepancy

The pilot study confirmed that athletes' self-evaluations and requirements vary across different self-states, resulting in self-discrepancies. Experiment 1 aimed to investigate whether tennis players with different levels of self-discrepancy differ in self-referential processing speed and self-descriptive judgment tendencies.

### 3.1 Method

#### 3.1.1 Participants

Based on the pilot study results and the calculation of self-discrepancy (Gabel et al., 2024), the top 20% and bottom 20% of tennis players were selected according to the normal distribution of self-discrepancy scores. Fourteen participants were assigned to the high self-discrepancy group (nine males, five females), and fourteen participants were assigned to the low self-discrepancy group (eight males, six females). Behavioral results from both groups were analyzed accordingly.

#### 3.1.2 Materials

The word stimuli used in Experiment 1 were identical to those in the pilot study, consisting of positive and negative words.

#### 3.1.3 Procedure

Participants classified as high or low self-discrepancy from the pilot study completed the self-referential paradigm after their tennis competitions. The experimental procedure and materials were consistent with the pilot study.

#### 3.1.4 Data analysis and processing

A mixed-design analysis of variance was conducted. Group (high vs. low self-discrepancy) was treated as a between-subjects variable, and word valence (positive vs. negative) was treated as a within-subjects variable. Reaction time (RT) and accuracy were used as dependent variables. Statistical analyses were performed using SPSS 27.0 (Field, 2024).

### 3.2 Results

#### 3.2.1 Self-referential processing speed

**Figure 2:**
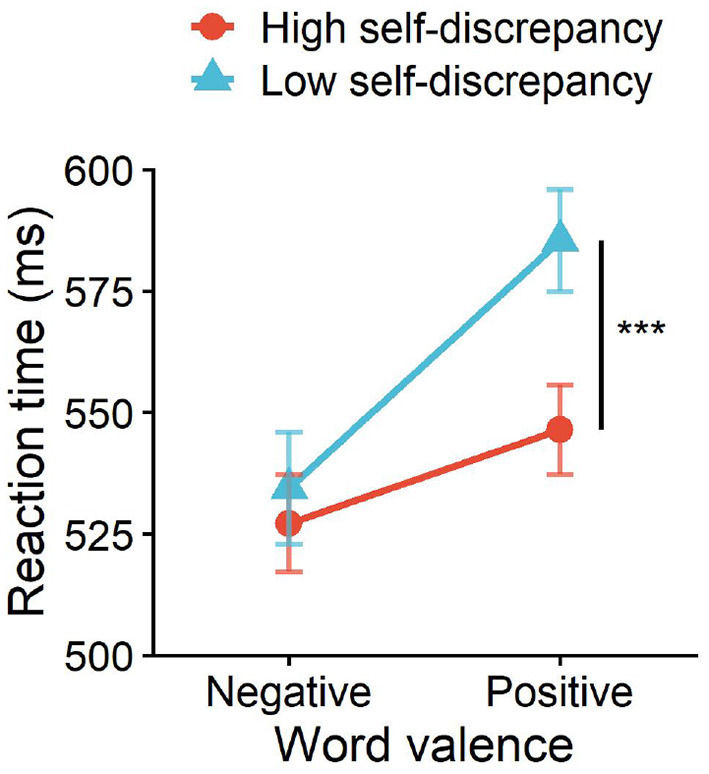
Interaction effect of self-discrepancy level and word valence on reaction time. ***p < 0.001.

For reaction times, the high self-discrepancy group responded to positive and negative words with mean RTs of 546.41 ± 34.59 and 527.16 ± 37.57 ms, respectively; the low self-discrepancy group responded with 585.40 ± 39.42 and 534.42 ± 43.13 ms. ANOVA results revealed a significant main effect of self-discrepancy level, *F*(1, 26) = 4.97, *p* = 0.030, $\eta_{p}^{2}$ = 0.16, and a significant interaction between word valence and self-discrepancy level, *F*(1, 26) = 11.46, *p* < 0.01, $\eta_{p}^{2}$ = 0.31. Simple-effects analyses revealed that for positive words, the high self-discrepancy group responded significantly faster than the low self-discrepancy group, *F*(1, 26) = 7.74, *p* = 0.010, $\eta_{p}^{2}$ = 0.23, whereas for negative words the group difference was small and non-significant, *F*(1, 26) = 0.23, *p* = 0.64, $\eta_{p}^{2}$ = 0.01, despite numerically shorter RTs in the high self-discrepancy group (see Figure 2).

#### 3.2.2 Self-descriptive judgments

**Figure 3:**
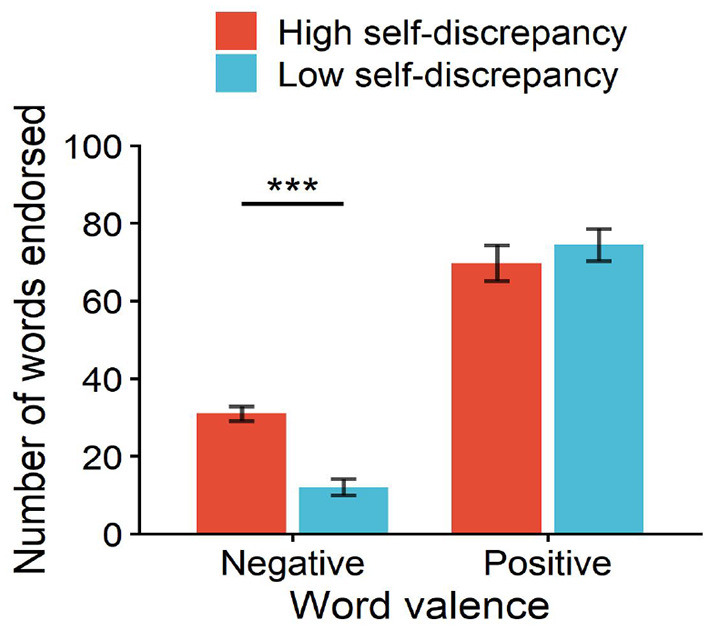
Interaction effect of self-discrepancy level and word valence on the number of words endorsed. ***p < 0.001.

For the number of words endorsed, the high self-discrepancy group selected 69.71 ± 17.38 positive words and 31 ± 7 negative words, while the low self-discrepancy group selected 74.43 ± 15.38 positive words and 12 ± 8 negative words. ANOVA results showed significant main effects of word valence, *F*(1, 26) = 220.02, *p* < 0.01, $\eta_{p}^{2}$ = 0.89, and self-discrepancy level, *F*(1, 26) = 4.39, *p* < 0.05, $\eta_{p}^{2}$ = 0.15, as well as a significant interaction, *F*(1, 26) = 12.10, *p* < 0.01, $\eta_{p}^{2}$ = 0.32. Simple-effects tests confirmed that for negative words, high self-discrepancy athletes endorsed significantly more items than low self-discrepancy athletes, *F*(1, 26) = 44.73, *p* < 0.001, $\eta_{p}^{2}$ = 0.63, whereas the groups did not differ significantly in the number of positive words endorsed, *F*(1, 26) = 0.58, *p* = 0.45, $\eta_{p}^{2}$ = 0.02 (see Figure 3).

### 3.3 Discussion

The results of this study indicate that self-discrepancy levels play a significant role in tennis players' cognitive processing and self-descriptive judgments.

First, regarding self-referential processing speed, participants in the high self-discrepancy group responded faster to negative words, suggesting greater sensitivity to negative information. This finding is consistent with Hypothesis 1 and aligns with previous research showing that individuals with anxiety or depressive tendencies exhibit faster processing of negative information. Extending beyond a descriptive pattern, these data support a schema-congruency account: when negative self-representations are chronically accessible, processing pipelines for congruent content become more efficient, shortening RTs for negative self-referent words while leaving positive-word RTs comparatively unchanged. Functionally, this implies that high self-discrepancy athletes may “arrive sooner” at negative self-meanings during evaluative moments in competition (e.g., after an error), increasing the likelihood of rapid—but potentially maladaptive—affective shifts.

Second, in terms of self-descriptive judgments, the high self-discrepancy group was more likely to describe themselves using negative words, while no significant difference was observed for positive words compared to the low self-discrepancy group. This supports Hypothesis 2 and is consistent with Higgins' (1987) Self-Discrepancy Theory. Importantly, endorsement asymmetry (greater negative than positive self-endorsement under high discrepancy) suggests that self-discrepancy is not merely an affective byproduct but operates as a content-selective filter shaping self-knowledge retrieval. This selectivity may stabilize a negative self-schema over time, thereby increasing the inertia of negative appraisals across training and competition cycles.

#### 3.3.1 Theoretical implications

Taken together, Experiment 1 links structural features of the self (discrepancy magnitude) to two separable components of self-referential processing—access speed and evaluative endorsement. The dissociation (RT vs. endorsement) suggests partially distinct mechanisms: rapid access may reflect early, automatic activation of schema-congruent representations, whereas endorsement reflects later, controlled evaluation stages. This layered view aligns with dual-process models of self-evaluation and clarifies why athletes can both “feel” negative meanings quickly and subsequently endorse them as self-descriptive.

#### 3.3.2 Ecological relevance

In tennis, self-evaluative episodes concentrate at changeovers and after critical points. Faster access to negative self-meanings can compress the temporal window for regulatory reappraisal, increasing the probability that the first, schema-consistent interpretation dominates subsequent behavior (e.g., conservative shot selection, risk aversion on second serves). Thus, Experiment 1 provides a plausible cognitive route from self-discrepancy to performance-relevant decisions.

#### 3.3.3 Alternative explanations and boundary conditions

Although group differences persisted after standard data cleaning, individual differences in trait anxiety or perfectionistic concerns could partially account for the negative-endorsement tilt. Future analyses including these covariates (or moderated models) would help disambiguate whether self-discrepancy uniquely predicts negative self-endorsement beyond shared variance with anxiety-related traits.

#### 3.3.4 Practical implications

Interventions that target the evaluative stage (e.g., cognitive restructuring) may attenuate negative endorsements, while attentional control or metacognitive drills may slow the “automatic capture” of negative meanings, widening the window for adaptive reappraisal between points.

#### 3.3.5 Limitations

Because Experiment 1 used lexical adjectives, generalization to richer, match-like stimuli remains to be shown; subsequent work should test whether the same RT/endorsement dissociation appears for video-based self-referent vignettes.

## 4 Experiment 2: characteristics of emotional information processing in tennis players with different levels of self-discrepancy

### 4.1 Method

#### 4.1.1 Participants

The participants in Experiment 2 were identical to those in Experiment 1: 14 athletes in the high self-discrepancy group and 14 athletes in the low self-discrepancy group.

#### 4.1.2 Materials

Emotional stimuli were selected from the Chinese Affective Picture System (CAPS), including 60 positive images, 60 negative images, and 130 neutral images. All images were uniformly processed using Adobe Photoshop, with a resolution of 260 × 300 pixels and a file size of 287 KB.

##### 4.1.2.1 Stimuli evaluation

Participants from the pilot study evaluated the emotional valence and arousal of the images using a 1–7 rating scale, consistent with the original CAPS evaluation procedure. Participants rated the images based on their immediate, subjective emotional responses, with no time constraints. Selection criteria for experimental materials were as follows: for arousal, positive and negative faces >4, neutral faces < 3; for valence, negative faces < 3, positive faces >5, neutral faces between 3 and 5.

One-way ANOVA showed that, in terms of arousal, positive and negative faces were rated higher than neutral faces (*p* < 0.05), with no significant difference between positive and negative faces (*p* > 0.05). In terms of valence, positive images were rated higher than negative images (*p* < 0.05), and neutral images were rated higher than negative images (*p* < 0.05). Based on these evaluations, experimental materials were composed as follows: 60 positive–neutral pairs, 60 negative–neutral pairs, and 125 neutral–neutral pairs, with five neutral–neutral pairs reserved for practice trials.

#### 4.1.3 Procedure

The experiment was conducted in a quiet lounge following the athletes' competitions. The task was programmed using E-prime 2.0 and presented on a laptop monitor with a visible area of 322 mm × 213 mm, a refresh rate of 60 Hz, and a resolution of 1,024 × 768 pixels. Participants were seated approximately 60 cm from the screen.

**Figure 4:**
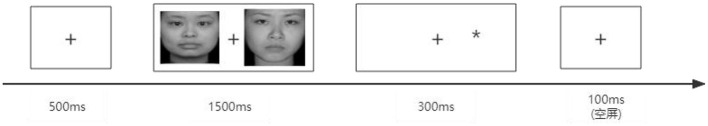
A dot-probe paradigm.

A dot-probe paradigm was employed. Each trial began with a fixation cross (“+”) presented at the center of the screen for 500 ms, followed by a randomly presented pair of images for 1,500 ms. After the images disappeared, a probe (“^*^”) appeared randomly at the location of one of the images for 300 ms. Participants responded to the probe location by pressing “F” for left or “J” for right. Trials without a response proceeded automatically after 3,000 ms. A black screen was displayed for 100 ms between trials (see Figure 4).

The experiment included 180 image pairs, with each emotional pair presented once, resulting in 180 trials in total. Before the formal task, participants completed five practice trials (neutral–neutral), which did not appear again in the formal experiment.

#### 4.1.4 Data analysis and processing

A three-factor mixed ANOVA was conducted with self-discrepancy group (high vs. low) as the between-subjects factor and picture emotion type (positive–neutral, negative–neutral, neutral–neutral) and cue type (probe congruent vs. incongruent with the emotional picture) as within-subjects factors. All analyses reported significance levels (*p* < 0.05) and corresponding effect sizes.

For the reaction time (RT) analysis, only correct trials from the formal task were included; practice trials were discarded. Trials with incorrect or missing responses were excluded from the dataset, and no further RT-based outlier trimming was applied. Across participants, these excluded trials accounted for approximately 5.3% of all trials, ensuring that the RT results were not driven by a small number of such errors.

Attentional-bias scores were then computed using a standard dot-probe convention. For each emotion condition (positive–neutral, negative–neutral), mean RTs were calculated separately for probes appearing at the same location as the emotional picture (congruent) and at the opposite location (incongruent), after applying all exclusions described above. The valence-specific bias index was defined as:


$$\begin{array}{l} \text{AB\_valence}=\text{RT\_incongruent}-\text{RT\_congruent}, \end{array}$$

such that larger positive values indicate greater vigilance toward the emotional picture. By design, the neutral–neutral condition yields AB ≈ 0. As an overall bias metric, we additionally computed a valence-differential index:


$$\begin{array}{l} \text{AB\_neg}-\text{pos}=\text{AB\_negative}-\text{AB\_positive}, \end{array}$$

with positive scores reflecting a relatively stronger attentional bias toward negative as compared to positive stimuli.

### 4.2 Results

#### 4.2.1 Emotional information processing speed

**Figure 5:**
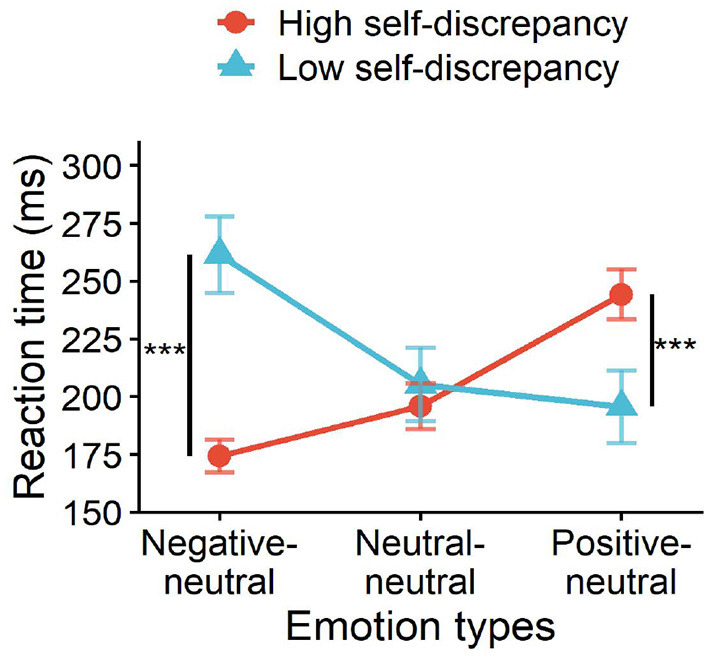
Interaction effect of self-discrepancy level and emotion type on reaction time. ***p < 0.001.

In the 2 (self-discrepancy level: high, low) × 3 (emotion type: negative–neutral, positive–neutral, neutral–neutral) mixed design, the main effect of self-discrepancy was not significant, *F*(1, 26) = 2.22, *p* = 0.141, $\eta_{p}^{2}$ = 0.08; the main effect of emotion type was also not significant, *F*(2, 52) = 1.30, *p* = 0.278, $\eta_{p}^{2}$ = 0.05. However, the interaction between emotion type and self-discrepancy level was significant, *F*(2, 52) = 13.43, *p* < 0.01, $\eta_{p}^{2}$ = 0.34. Simple effect analysis indicated that athletes in the high self-discrepancy group processed negative emotional stimuli significantly faster than those in the low self-discrepancy group, *F*(1, 26) = 3.55, *p* = 0.002, $\eta_{p}^{2}$ = 0.12. In contrast, for positive emotional stimuli, the high self-discrepancy group responded more slowly than the low self-discrepancy group, while no significant difference was observed for neutral stimuli (see Figure 5).

#### 4.2.2 Attentional bias

**Figure 6:**
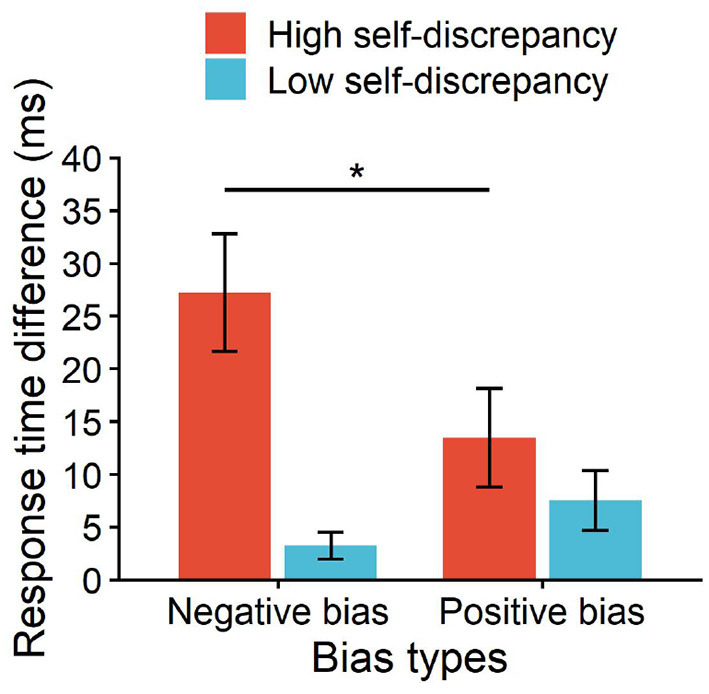
Differences in attentional bias between high and low self-discrepancy tennis players under positive and negative picture conditions. *p < 0.05.

Under the negative picture condition, the attentional bias score was 27.21 for the high self-discrepancy group and 3.25 for the low self-discrepancy group, with a significant difference (*p* < 0.01). Under the positive picture condition, the attentional bias score was 13.46 for the high self-discrepancy group and 7.53 for the low self-discrepancy group, with no significant difference (*p* = 0.293). Further analysis revealed that the high self-discrepancy group showed significant differences between negative and positive attentional bias, *F*(1, 26) = 2.46, *p* = 0.017, $\eta_{p}^{2}$ = 0.09, whereas the low self-discrepancy group did not show significant differences (*p* > 0.05) (see Figure 6).

#### 4.2.3 Cue congruency effect

A 2 (self-discrepancy level: high, low) × 2 (cue type: congruent, incongruent) × 2 (emotion type: positive–neutral, negative–neutral) three-way ANOVA was conducted. The main effect of self-discrepancy was significant, *F*(1, 26) = 8.32, *p* = 0.005, $\eta_{p}^{2}$ = 0.24; the main effect of cue type was also significant, *F*(1, 26) = 360.14, *p* < 0.01, $\eta_{p}^{2}$ = 0.93; and the three-way interaction was significant, *F*(1, 26) = 46.25, *p* < 0.01, $\eta_{p}^{2}$ = 0.64.

Under cue-congruent conditions, the high self-discrepancy group (*M* = 136.35, *SD* = 31.30) responded faster than the low self-discrepancy group (*M* = 169.84, *SD* = 32.56).

Under cue-incongruent conditions, the high self-discrepancy group (*M* = 265.68, *SD* = 24.16) responded more slowly than the low self-discrepancy group (*M* = 206.90, *SD* = 32.79).

**Figure 7:**
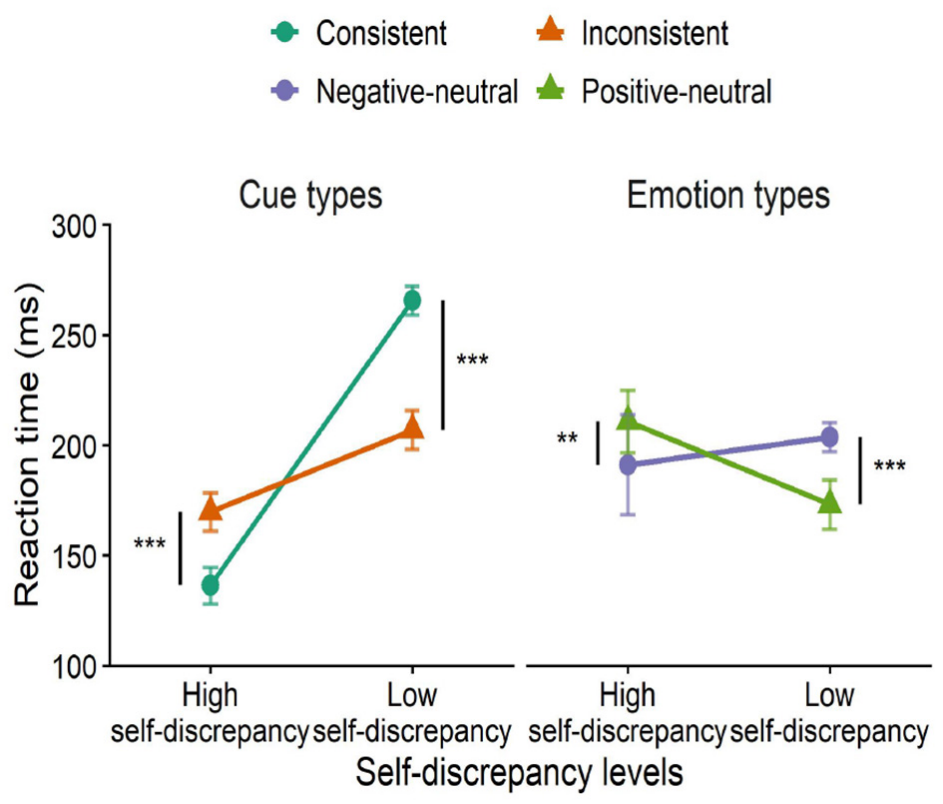
Interaction effects of self-discrepancy level, cue congruency, and emotion type on reaction time. **p < 0.01, ***p < 0.001.

In terms of emotion type, the high self-discrepancy group processed negative stimuli faster (*M* = 191.18, *SD* = 84.97), whereas the low self-discrepancy group processed positive stimuli faster (*M* = 173.13, *SD* = 41.88) (see Figure 7).

### 4.3 Discussion

The findings of this study demonstrate that self-discrepancy levels significantly influence athletes' performance in emotional information processing.

First, regarding reaction times, athletes with high self-discrepancy responded more quickly to negative emotional stimuli, whereas those with low self-discrepancy responded faster under positive emotional conditions. Moving beyond description, this pattern indicates a valence-specific tuning of early orienting systems consistent with vigilance for threat among high-discrepancy athletes. In competitive play, such tuning can prioritize detection of adverse signals (e.g., opponent momentum shifts), but at the cost of reduced responsiveness to appetitive or recovery-promoting cues, potentially prolonging negative affect after errors.

Second, in terms of attentional bias, the high self-discrepancy group exhibited a significantly stronger negative bias. Process-wise, dot-probe indices point to two candidate components: (a) facilitated orienting toward negative locations; and (b) impaired disengagement once attention is captured. The robust cue-congruency effects in our data—especially the disproportionate slowing on incongruent trials for high-discrepancy athletes—favor the disengagement account, implying “sticky” attention to negative cues. This stickiness is performance-relevant, because difficulty disengaging can delay a return to task-focused information (tactical plans, serve routines).

Third, cue congruency analysis further revealed an interaction pattern between self-discrepancy level and emotional cue processing. Crucially, faster responses under congruent–negative conditions alongside marked costs under incongruent conditions suggest an asymmetry: negative cues help high-discrepancy athletes when the upcoming probe location matches their vigilance set, but harm them when rapid shifting is required. In tennis, this asymmetry maps onto momentum situations—rapid exploitation of negative signals (e.g., sensing opponent pressure) vs. sluggish reorientation when positivity or neutrality is behaviorally optimal (e.g., committing to a courageous first-serve target after a double fault).

#### 4.3.1 Integration with Experiment 1

Experiment 2 complements the lexical self-referential findings by demonstrating that the same self-structural factor (discrepancy) extends to spatial attention. Together, the studies outline a pipeline: self-discrepancy → faster access to negative self-meanings (Exp. 1) → preferential orienting and reduced disengagement from negative cues in space (Exp. 2). This cross-task convergence strengthens the claim that self-discrepancy shapes both representational and attentional layers of emotional processing.

#### 4.3.2 Applied implications

For high-discrepancy athletes, attention-bias modification protocols emphasizing disengagement from negative locations, combined with serve-routine elements that force a brief neutral focus (breath count, tactile cue), may counteract “sticky” negative capture. Coaches can also structure drills where athletes must rapidly shift from processing negative feedback to executing a preplanned, positively framed action (e.g., “error → reset cue → commit to aggressive target”).

#### 4.3.3 Alternative explanations and controls

Although groups were matched by design, arousal differences among image categories could inflate orienting effects. Nonetheless, the interaction pattern (valence × congruency × group) is difficult to explain by arousal alone. Including trial-level arousal ratings or pupilometry in future work would allow explicit partitioning of valence and arousal contributions.

#### 4.3.4 Limitations and future directions

Behavioral indices cannot localize neural loci of disengagement costs. Future studies should integrate ERP markers (e.g., N2pc for orienting, Pd for distractor suppression; LPP for sustained affective processing) to test whether high-discrepancy athletes show amplified sustained attention to negative cues or attenuated suppression signals during reorienting. Field-valid tasks (video-based rally sequences; *in-situ* eye-tracking during serve routines) will further establish ecological validity.

## 5 General discussion

Through two experiments, this study systematically revealed differences in emotional information processing among tennis players with varying levels of self-discrepancy. First, Experiment 1 found that athletes with high self-discrepancy responded faster to negative information and were more inclined to use negative words in self-description, whereas those with low self-discrepancy focused more on positive information. This pattern aligns with the self-referential cognitive model: when individuals are in a negative emotional state, they automatically attend to and process congruent negative information, thereby accelerating negative processing. Our findings support Beck's schema theory, which proposes that individuals with high self-discrepancy are prone to forming negative cognitive schemas and preferentially process negative self-relevant information (Yang, 2015; Hu et al., 2022; Bargh and Tota, 1988).

Second, Experiment 2 further demonstrated that athletes with high self-discrepancy displayed heightened vigilance toward negative faces in visuospatial attention. They responded rapidly when probes appeared on negative–neutral face pairs, but showed slower reactions under positive conditions. This indicates that self-discrepancy alters the allocation of attentional bias components—orienting and vigilance become the primary mechanisms underlying this bias. As prior studies have shown, highly anxious individuals are more likely to exhibit vigilance toward negative stimuli while neglecting positive cues (Klumpp and Amir, 2009; Abado et al., 2020; Richards et al., 2014). Our findings extend this conclusion by demonstrating that self-discrepancy similarly drives biased emotional attention patterns in a non-clinical athlete population.

Theoretically, this study demonstrates the applicability of self-discrepancy theory in the field of sport psychology. Self-discrepancy can be considered a latent self-schema that, once activated, enhances negative bias in emotional information processing. Martín-García et al. (2024) also noted that self-discrepancy influences individuals' interpretation of ambiguous situations by activating negative processing modes, thereby contributing to anxiety and depressive symptoms. Our data are consistent with this: athletes with high self-discrepancy exhibited a stronger tendency toward negative processing and may thus be more vulnerable to negative emotional experiences.

From a practical perspective, these findings highlight the importance of monitoring athletes' self-evaluations in psychological assessment and training. For athletes with high self-discrepancy, interventions such as cognitive restructuring or attention bias modification training (ABMT) could help adjust information processing patterns and mitigate maladaptive emotional effects (Zhao et al., 2025; Heeren et al., 2015).

Nevertheless, this study has several limitations. The sample size was relatively small and restricted to athletes in Hunan Province, limiting generalizability. The experimental tasks were conducted in a laboratory setting, which may not fully capture real competition scenarios. Moreover, analyses of attentional bias were based solely on behavioral data; future studies could integrate neurophysiological measures such as event-related potentials (ERP) or functional magnetic resonance imaging (fMRI) to explore the underlying neural mechanisms. Further research should also expand the participant pool to include athletes from different sports and performance levels, and examine the efficacy of training programs designed to reduce negative attentional bias.

## 6 Conclusion

The level of self-discrepancy significantly influences tennis players' emotional information processing characteristics. Athletes with higher self-discrepancy are more likely to form negative cognitive schemas, leading to faster negative processing and a pronounced negative attentional bias during emotional processing. These findings underscore the critical role of individual differences in self-cognition for athletes' emotional regulation and provide a novel perspective for research in sport psychology.

## Data Availability

The original contributions presented in the study are included in the article/supplementary material, further inquiries can be directed to the corresponding author.

## References

[B1] Abado E. Richter T. Okon-Singer H. (2020). “Attention bias toward negative stimuli,” in Cognitive Biases in Health and Psychiatric Disorders (Academic Press), 1940. doi: 10.1016/B978-0-12-816660-4.00002-7

[B2] Bargh J. A. Tota M. E. (1988). Context-dependent automatic processing in depression: accessibility of negative constructs with regard to self but not others. J. Pers. Soc. Psychol. 54, 925–939. doi: 10.1037/0022-3514.54.6.925 3397867

[B3] Bar-Haim Y. Lamy D. Pergamin L. Bakermans-Kranenburg M. J. van IJzendoorn M. H. (2007). Threat-related attentional bias in anxious and nonanxious individuals: a meta-analytic study. Psychol. Bull. 133, 1–24. doi: 10.1037/0033-2909.133.1.1 17201568

[B4] Brèdart S. (2016). A self-reference effect on memory for people: we are particularly good at retrieving people named like us. Front. Psychol. 7:1751. doi: 10.3389/fpsyg.2016.01751 27881969 PMC5101417

[B5] Bryant R. A. (2021). Post-traumatic stress disorder: a state-of-the-art review of evidence and challenges. World Psychiatry 20, 259–269. doi: 10.1002/wps.20656 31496089 PMC6732680

[B6] Eysenck M. W. Derakshan N. Santos R. Calvo M. G. (2007). Anxiety and cognitive performance: attentional control theory. Emotion, 7, 336–353. doi: 10.1037/1528-3542.7.2.336 17516812

[B7] Field A. (2024). Discovering Statistics Using IBM SPSS Statistics. Los Angeles, CA: Sage Publications Limited.

[B8] Gabel L. N. Olino T. M. Goldstein B. L. Klein D. N. Stanton K. Hayden E. P. . (2024). Latent structure and item functioning of self-referent encoding task word stimuli in preadolescent youth. Assessment 32, 1103–1119. doi: 10.1177/10731911241289249 39579042 PMC12397560

[B9] Gürcan-Yıldırnm D. Gençöz T. (2022). The association of self-discrepancy with depression and anxiety: moderator roles of emotion regulation and resilience. Curr. Psychol. 41, 18211834. doi: 10.1007/s12144-020-00701-8

[B10] Heeren A. Mogoaşe C. Philippot P. McNally R. J. (2015). Attention bias modification for social anxiety: a systematic review and meta-analysis. Clin. Psychol. Rev. 40, 76–90. doi: 10.1016/j.cpr.2015.06.001 26080314

[B11] Heron K. E. Smyth J. M. (2013). Body image discrepancy and negative affect in women's everyday lives: an ecological momentary assessment evaluation of self-discrepancy theory. J. Soc. Clin. Psychol. 32, 276–295. doi: 10.1521/jscp.2013.32.3.276

[B12] Higgins E. T. (1987). Self-discrepancy: a theory relating self and affect. Psychol. Rev. 94, 319–340. doi: 10.1037/0033-295X.94.3.319 3615707

[B13] Hu C. Cao R. Huang J. Wei Y. (2022). The effect of self-discrepancy on online behavior: a literature review. Front. Psychol. 13:883736. doi: 10.3389/fpsyg.2022.883736 35558697 PMC9087717

[B14] Jin Y. He J. (2021). Effects of visual search task on attentional bias and stress response under pressure. Work 69, 687–696. doi: 10.3233/WOR-213509 34120945

[B15] Klumpp H. Amir N. (2009). Examination of vigilance and disengagement of threat in social anxiety with a probe detection task. Anxiety Stress Coping 22, 283296. doi: 10.1080/10615800802449602 19253172 PMC3712328

[B16] Kujawa A. Kessel E. M. Burkhouse K. L. Hajcak G. (2022). Neural and cognitive mechanisms of self-referential processing in emotional disorders. Cogn. Affect. Behav. Neurosci. 22, 456–470.

[B17] MacLeod C. Mathews A. Tata P. (1986). Attentional bias in emotional disorders. J. Abnorm. Psychol. 95, 15–20. doi: 10.1037/0021-843X.95.1.15 3700842

[B18] Martín-García O. Blanco I. Sánchez-López Á. (2024). Do we interpret ambiguity and feel according to how we define ourselves? Relationships between self-perception, interpretation biases, and their role on emotional symptoms. Front. Psychiatry 15:1502130. doi: 10.3389/fpsyt.2024.1502130 39758451 PMC11695330

[B19] Mogg K. Bradley B. P. (1998). A cognitive-motivational analysis of anxiety. Behav. Res. Ther. 36, 809–848. doi: 10.1016/S0005-7967(98)00063-1 9701859

[B20] Oh H. Lee D. Cho H. (2023). The differential roles of shame and guilt in the relationship between self-discrepancy and psychological maladjustment. Front. Psychol. 14:1215177. doi: 10.3389/fpsyg.2023.1215177 37842708 PMC10573311

[B21] Perera A. T. M. Sharma I. Stephen I. D. (2025). Dot probe tasks produce no attentional modifications towards healthy weight bodies. Eur. Eat. Disord. Rev. 34, 281–287. doi: 10.1002/erv.70028 40879213

[B22] Prior E. Papathomas A. Rhind D. (2025). A balancing act: sport psychologist insights into supporting athlete mental health in elite sport. J. Appl. Sport Psychol. 37, 701–721. doi: 10.1080/10413200.2025.2462551

[B23] Rahimi A. Roberts S. D. Baker J. R. Wojtowicz M. (2022). Attention and executive control in varsity athletes engaging in strategic and static sports. PLoS ONE 17:e0266933. doi: 10.1371/journal.pone.0266933 35452468 PMC9032374

[B24] Richards H. J. Benson V. Donnelly N. Hadwin J. A. (2014). Exploring the function of selective attention and hypervigilance for threat in anxiety. Clin. Psychol. Rev. 34, 1–13. doi: 10.1016/j.cpr.2013.10.006 24286750

[B25] Sui J. Humphreys G. W. (2015). The integrative self: How self-reference integrates perception and memory. Trends cogn sci, 19(12), 719–728. doi: 10.1016/j.tics.2015.08.015 26447060

[B26] Wang Y. Zhou L. Luo Y. (2008). The pilot establishment and evaluation of Chinese affective words system. Chinese Ment. Health J. 22, 608–612. doi: 10.3321/j.issn:1000-6729.2008.08.014

[B27] Xu N. Zhang X. Liu X. Sun M. Rui L. Wang Y. (2022). Attentional biases toward face-related stimuli among athletes after state thwarting of need for relatedness. Comput. Intell. Neurosci. 2022:2491051. doi: 10.1155/2022/2491051 35586109 PMC9110145

[B28] Yang H. (2015). Psychological resilience in college students: relationships among stressful life events, self-discrepancy, social support, positive coping, and school adjustment (Doctoral dissertation). Southwest University, Chongqing, China.

[B29] Yaoi K. Osaka M. Osaka N. (2015). Neural correlates of the self-reference effect: evidence from evaluation and recognition processes. Front. Hum. Neurosci. 9:383. doi: 10.3389/fnhum.2015.00383 26167149 PMC4481146

[B30] Zhang Y. Zhong Y. (2012). The regulatory role of attentional resources on negative bias under different self conditions. J. Xianning Univ. 32, 79–83.

[B31] Zhao J. Yang Y. Wang Q. Zhang H. Nie Y. (2025). Effect of attentional bias modification on pre-competition anxiety in athletes. Front. Psychol. 16:1596298. doi: 10.3389/fpsyg.2025.1596298 40918283 PMC12409969

[B32] Zhao S. Uono S. Hu R. Q. Yoshimura S. Toichi M. (2023). Self-referential and social saliency information influences memory following attention orienting. Front. Psychol. 14:1092512. doi: 10.3389/fpsyg.2023.1092512 37034947 PMC10075135

[B33] Zhao Y. Xu J. Hong J. Xu X. Fan H. Zhang J. . (2024). Behavioral evidence of impaired self-referential processing in patients with affective disorders and first-episode schizophrenia. Sci. Rep. 14:10754. doi: 10.1038/s41598-024-60498-5 38730229 PMC11087487

